# Unravelling high insect diversity and community turnover along a tropical-temperate elevation gradient: A metabarcoding approach

**DOI:** 10.1371/journal.pone.0327884

**Published:** 2025-07-17

**Authors:** Damián Villaseñor-Amador, Pilar Benites, Fatima M. Sandoval-Becerra, Madai Rosas-Mejía, Alejandro Zaldívar-Riverón, Milan Janda

**Affiliations:** 1 Deparment of Zoology, Faculty of Science, Palacky University, Olomouc, Czech Republic; 2 Conservación de Anfibios A.C., Puebla, Mexico; 3 Colección Nacional de Insectos, Instituto de Biología, Universidad Nacional Autónoma de México, Ciudad de México, Mexico; 4 Laboratorio Nacional de Análisis y Síntesis Ecológica, Escuela Nacional de Estudios Superiores Unidad Morelia, Universidad Nacional Autónoma de México, (UNAM), Morelia, Michoacán, Mexico; 5 Instituto de Ecología Aplicada, Universidad Autónoma de Tamaulipas (UAT), Ciudad Victoria, Tamaulipas, Mexico; 6 Facultad de Medicina Veterinaria y Zootecnia, Universidad Autónoma de Tamaulipas, Ciudad Victoria, Tamaulipas, Mexico; 7 Escuela Nacional de Estudios Superiores, Universidad Nacional Autónoma de México Morelia, Michoacán, Mexico; 8 Biology Centre of Czech Academy of Sciences, Ceske Budejovice, Czech Republic; MARE – Marine and Environmental Sciences Centre, PORTUGAL

## Abstract

The transition zone between the Nearctic and Neotropical biogeographic regions is one of the most species-rich areas of North America, known as the Mexican Transition Zone. We sampled mobile insects along a 2000 m elevational gradient for 13 months using flight interception traps (Malaise) to evaluate their diversity, community structure and environmental factors linked to their distribution. We identified 3091 Molecular Operational Taxonomical Units (560 ± 199 SD per trap), out of which 513 were identified to genus and 197 to species. Our results show high turnover at both species and genus levels across the elevational gradient. Elevational diversity patterns varied across taxa: Coleoptera and Lepidoptera showed their highest diversity at mid-elevations, while Diptera and Hymenoptera had increased diversity with elevation. Temperature and vegetation composition best explained the spatial fluctuations of insect diversity. Our work represents the most comprehensive survey of insect communities in the region to date. By combining a long-term survey with high-throughput metabarcoding, this study provides an overview of regional diversity and establishes a foundation for detailed follow-up studies.

## Introduction

Mountains represent valuable systems for ecological studies due to the steep biotic and abiotic transitions they encompass over relatively small spatial scales [1]. These gradients offer unique opportunities to investigate species distributions [2] and to explore patterns of diversity and community structure dynamics [3–6]. Elevational gradients in particular support high biodiversity [2,7], with tropical mountains hosting some of the world’s highest densities of terrestrial organisms [8]. However, biodiversity research has historically been biased towards plants [9] and vertebrates [10–12], leaving invertebrate communities underrepresented.

In most terrestrial ecosystems, insects likely constitute the largest proportion of animal biomass [13]. Elevational stratification is a common feature among various insect taxa, including ants [14–16], moths [4,6], and beetles [17]. However, different insect groups exhibit distinct responses to elevational gradients. Some species are restricted to narrow altitudinal ranges, resulting in high turnover rates and significant levels of endemism [18,19], while others tolerate a broad range of environmental conditions across entire elevational gradients [19]. Consequently, the relationship between insect richness and elevation varies, with patterns ranging from linear declines [20] to unimodal or hump-shaped distributions [14,16]. The hump-shaped pattern, in particular, is often used as a null model for understanding species richness along altitudinal gradients, as it reflects the random distribution of species ranges within a given geographical domain, a phenomenon known as the mid-domain effect [11,21]. Alternatively, environmental factors may drive the diversity pattern of the elevational gradient, particularly well-known abiotic predictors such as temperature or rain [10]. Additionally, these distributional patterns are also influenced by the specific life history and morphological traits of insect groups [22].

Flying insects tend to be more abundant and species-rich at mid elevations, with their numbers peaking at mid-elevations (>500 m), before declining [4,6]. In contrast, ground-dwelling insects, such as beetles, often show a hump-shaped distribution of abundance and species richness with increasing elevation [17,23,24]. However, other leaf litter and soil-dweling groups, such as ants, frequently show exponential declines in abundance with increasing latitude [14]. Despite these general trends, insect elevational diversity patterns across broad versus narrow taxonomic categories remain relatively unexplored. For example, few studies have assessed the relationship between genus richness and elevation or compared species- and genus-level turnover along elevational gradients [25]. Investigating these patterns in biodiversity-rich-regions could provide valuable insights into the ecological factors shaping insect distribution and diversity.

Here, we analysed insect assemblage richness and composition along an elevational gradient in El Cielo Biosphere Reserve, México, using Malaise trapping during 13 consecutive months. This reserve is situated within the Mexican Transition Zone, a biogeographical region where biotas of different evolutionary origins converge and overlap [26]. The aims of our study were to: (a) characterise insect diversity and community composition across different elevations in this tropical-temperate transition zone, and (b) evaluate the environmental factors influencing insect assemblages along the gradient.

Based on previous insect richness studies along elevation gradients [14,16], we hypothesised that insect richness follows a hump-shaped pattern, primarily driven by the mid-domain effect or alternatively, by environmental factors known to determine diversity patterns along elevational gradients (ex. temperature and rainfall [10]). We suggest that the highest levels of richness occur at mid-elevations due to the overlap of species’ elevational ranges. We also anticipated that diversity along the gradient would mainly result from species turnover (replacement) and nestedness (species gains and losses). Environmental factors such as temperature and humidity, which change predictably with altitude, were expected to be major constraints shaping the distribution and richness of insect species [6,10]. Our long-term study represents the most comprehensive analysis to date of the relationship between elevation and insect diversity in the Mexican Transition Zone, a region of high biological significance.

## Methods

### Ethics statement

Field research in El Cielo Biosphere Reserve was approved under permit No. SGPA/DGVS/03187/20 issued by México’s Secretariat of Environment and Natural Resources. The insect species used in this study are not protected and therefore no further species-specific permits were requested for sampling.

### Study area

El Cielo Biosphere Reserve (hereafter El Cielo) is situated in the Sierra Madre Oriental biogeographic province [26], in northeastern Mexico (23.25N, − 99.833W). It covers 1445.3 square km with a maximum elevation of 2320 m of altitude [27]. Vegetation gradient includes deciduous and semideciduous tropical forests at 150–800 m, cloud forests at 800–1500 m, oak-pine forests at 1200–1900 m and pine forests at 1800–2200 m [28]. The studied mountain within El Cielo hosts a tropical–temperate transitional primary forest, with vegetation that shifts from plants with tropical origin at the lower elevations [28] to plants with temperate affinities in mid and higher elevations, such as *Quercus* and *Pinus* species [29].

### Study design and data collection

An elevational transect was established in the municipality of Gómez Farías, Tamaulipas, Mexico, in 2019 and 2020. The transect extended east to west from 23.0369N, − 99.1316W to 23.0414N, − 99.2671W, covering an approximately aerial distance of 14 km. Malaise traps were deployed at elevations ranging from 200 to 1800 m (S1 Fig) at 200 m intervals, with nine traps placed in conserved habitats with vegetation representative of each elevation. The traps were positioned in the forest understory, touching the soil, either beneath or near a large canopy tree, ensuring at least partial exposure to open areas to enhance the capture of flying insects.

Samples were collected from August 19, 2020, to September 29, 2021. Each trap was checked and refilled with ethanol every two weeks (14-day intervals) over a 13-month period. In total, we collected 234 samples representing nine elevations, of which 117 were meta-barcoded (corresponding to one sample per elevation per month). The remaining 117 samples were preserved for future taxonomic identification. Specimens were preserved in 95% non-denatured ethanol and stored in ziploc^®^ bags until further processing. The collecting bottles on the traps were covered with aluminium foil to prevent alcohol evaporation and degradation. During periods of heavy rain, the traps were checked, with ethanol being replaced as needed, and specimens were later pooled to align with the standard 14-day sampling intervals.

Environmental data were collected near the Malaise traps and inferred from available databases. Data on altitude, diameter at breast height (DBH), abundance, and richness of trees taller than 3 meters were collected within two 20 x 20 m plots at each elevation, set near the Malaise trap (~15 m). These plots were also used for parallel ecological surveys of selected insect groups. Local temperature and rainfall data were obtained from a meteorological station in Gómez Farías, provided by the Mexican Government’s National Meteorological Service (https://smn.conagua.gob.mx/). Additionally, long-term climate data (30-year period), including Annual Mean Temperature (BIO1) and Annual Precipitation (BIO12), as well as elevation data for map plotting, were retrieved from WorldClim at the highest available resolution (~1 km^2^) [30].

### Laboratory processing

We used metabarcoding to assess insect diversity along the elevation gradient. This technique has proved to be a reliable, verifiable and efficient method for monitoring biodiversity at a community level, especially in hyperdiverse and complex taxa, like arthropods [31]. Recent metabarcoding studies have yielded robust insect diversity assessments comparable to those that have employed traditional taxonomic approaches [32–35].

Samples were ethanol-drained and left to dry overnight at room temperature. Once completely dried, they were individually ground into fine powder using an Ultra Turrax Tube Drive grinder (IKA®) with sterile disposable tubes (IKA®) and 10 steel beads. We placed 25 mg of fine powder of each sample into a 1.5 ml tube, and all samples were subsequently sent to the Canadian Center for DNA Barcoding in the Guelph University, Ontario, Canada for laboratory processing.

DNA extraction was performed using commercial silica-columns kits. During powder sampling, an empty 1.5 tube with 200 µl ATL buffer was left open and replaced every 60 samples to control for cross-contamination due to the powder’s volatile nature (DNA extraction control), and processed in the same way as all samples down to sequencing. Lysis was performed overnight at 56ºC with 180 μL of ATL buffer and 20 μL of proteinase K. The supernatant was placed in silica columns and left incubating for 15 minutes, after which DNA was eluted in 80 μL of AE buffer. A second elution was performed after 5 minutes of incubation. Final products were quantified using Qubit® 2.0 fluorometer (Invitrogen) and eluted samples were diluted to 2 ng/ μL.

A two-step PCR amplification of the extracted DNA targeted a 418 bp COI segment using the arthropod BF3 and BR2 primers [36]. The first (PCR1) amplified the targeted BF/BR2 fragment, and the second (PCR2) added P5/P7 Illumina adapters with individual identification barcodes (6 to 8 bp) attached to them to maximize multiplexing. PCR1 had an initial denaturation of 95 °C for 5 min, followed by 30 cycles of 94°C for 30 s, 46°C for 30 s, and 72°C for 50 s, with a final extension at 72°C for 5 min. PCR2 had an initial denaturation of 2 m, followed by 15 cycles of 94°C for 40 sec, 51°C for 1 min, and 72°C for 1 min, with a final extension of 72°C for 5 min.

Each PCR reaction had volume of 25 µl composed of 2.5 μL of buffer (10X), 0.5 μL of dNTPs (10 mM), 1 μL of each forward and reverse primers (10 mM), 0.2 μL of mi-Taq (5 U/μL), 16.8 μL of extra pure molecular grade water, and 2 μL of DNA or 1x diluted PCR1 template. A negative control containing reaction mix without DNA (PCR control) was included every 96 subsamples.

For each sample, three technical replicates were PCR-amplified and sequenced in 96-well plates with three different identification barcodes attached to them to allow independent tracking of each PCR product after amplification and detect potential contamination. PCR products were purified using magnetic beads (ratio 0.8:1 μL beads/product) and pair-end sequenced using Illumina NovaSeq 6000 instrument.

### Sequencing reads processing and taxonomic assignment

The resulting reads were removed of any remaining adapters, cleaned and quality-filtered using AdapterRemoval v.2.3.0 and Sickle v.1.33. Sequence errors were corrected using Bayes-Hammer via SPAdes 3.12.0 and paired-end reads were merged using PANDAseq v.2.11. Reads were demultiplexed into samples using *sort.py* from the program DAMe v.1.0. PCR technical replicates per sample were filtered using *filter.py* from DAMe, and only reads present in all 3 PCRs with abundance equal or larger than 10 reads were retained in the final output. The quality of filtering and trimming was visually evaluated using FASTQC, and chimeric sequences were identified and removed through VSEARCH. Finally, reads were clustered into Molecular Operational Taxonomic Units (henceforth MOTUs) using SUMACLUST v.1.0.36, with a 97% similarity threshold. A MOTU is a categorical nominal variable representing an independent evolutionary unit, which corresponds to a presumptive species [37]. Even without formal species names, using MOTUs to describe biodiversity at community level has proven a viable alternative to the traditional taxonomy, which often registers only a fraction of the study site’s diversity, due to time, cost and taxonomic expertise constrains, which are especially scarce for invertebrate studies [38]. Results were filtered for erroneous MOTUs based on co-occurrence in the samples using the LULU package in R. Taxonomic identification was performed by comparing nucleotide sequences to the BOLD Systems database (identification hits) using the BOLD package v.1.3.0 in R and the BOLD_NCBI_Merger. Taxonomic assignment was kept only at the taxonomic level with no ambiguities. If a MOTU had multiple identifications at a particular taxonomic level (i.e., a nucleotide sequence matched multiple different identifications in the BOLD database), a unique and unambiguous identification at a higher level was retained (i.e., at the taxonomic level where all nucleotide sequences yielded identical results). We retained taxonomic identification at the family, genus and species levels for MOTUs with a similarity of 85%, 95% and 97%, respectively. Identifications with a similarity < 85% were discarded.

### Statistical analyses

#### Insect diversity and community composition.

To assess inventory completeness, sample-size-based, coverage-based, and sample completeness rarefaction/extrapolation curves were performed [39,40]. Species richness (Hill’s number: q = 0) was calculated using incidence frequency data, and we used this incidence frequency as our main response variable for posterior analyses. Insect incidence frequency can be understood as how many times each MOTU were seen in each of the sampling events (i.e. each of the 13 sampling months). To obtain this variable, a presence/absence matrix was created by pooling the MOTUs from each of the 117 samples collected and subsequently transforming all data into ones (presences) and zeros (absences). We also created a matrix for the frequency of occurrence in the month: each column represented an altitude (n = 9) and each row represented a MOTU. However, in contrast with the P/A matrix the frequency of occurrence matrix contained values that could range from 0–13 occurrences: zero if no specimen of that particular MOTU was caught at that elevation in the 13 months of sampling, or 13 if a specimen was caught in each of the 13 months of sampling. Site sample coverage ranged from 0.98 (200 m elevation) to 0.997 (400 m). The minimum sample coverage was above 0.9, so richness was not standardised for subsequent analyses. All sample coverage calculations were performed with a Hill number of q = 0, 40 knots, 95% confidence intervals and 99 bootstraps in the statistical software R v.4.3.2 [41] with the iNEXT v.2.0.20 package [40].

#### Environmental variables.

To assess the effect of environmental factors on the diversity patterns across the elevational gradient, we performed a Generalized Linear Model (GLM) using insect incidence frequency as the response variable and the following environmental predictors as explanatory variables: altitude, tree DBH, tree abundance, tree richness, temperature and rainfall. Specifically, for the incidence frequency response variable, we summed all the presences of each MOTU of every sample per altitude, to yield a vector of 9 values (one per each elevation). For the environmental explanatory variables, we calculated its average for each of the nine elevations. We tested for multicollinearity between the environmental variables and only chose those with a VIF < 1.5 [42] and a Spearman correlation coefficient *P* > 0.05. We used the *vifstep* function of the R package *usdm* v.2.1–7 [43] and the *chart.Correlation* function in the R package *PerformanceAnalytics* v.2.0.4 [44]. Selected variables were tree richness and Annual Mean Temperature. We assumed a Poisson distribution error for the GLM and performed a stepwise backward model simplification [45]. Only the effect of single explanatory variables and two-way interactions between explanatory variables were examined.

To test for seasonal fluctuations of temperature and rainfall across our study sites, we made a table similar to the month-trap incidence frequency matrix used to test the inventory completeness: each column stood for an altitudinal band (n = 9), and each row for a month (n = 13). Each cell value was the sum of all MOTU specimens caught for that specific altitude in that month. Additionally, an environmental predictors matrix was built, with 13 rows (13 months) and four columns: historic temperature (1991–2020), historic rainfall (1991–2020), contemporary temperature (2020–2021) and contemporary rainfall (2020–2021). Using these two matrices, we performed a model-based ordination with the *ecoCopula* R package [46] and its corresponding multivariate abundance analysis with the *mvabund* R package [47]. To obtain the explained variation by temperature and rainfall, we calculated the difference of the sum of absolute values of correlation coefficients between null models and models, including the environmental predictors [48].

#### Elevational diversity patterns.

To evaluate the relationship between elevation and species richness of insect communities, we used linear, quadratic, cubic, exponential and null models and compared those that best fitted the data [23]. Richness per trap was used (n = 9) as a response variable. The best fitted model for each insect order had the smallest delta Akaike Information Criterion for small samples (ΔAICc) and a normal distribution of residuals (Shapiro–Wilk test, *p* > 0.05) [49]. Insect orders with different best-fit models (linear, quadratic, cubic, exponential) were determined to follow different altitudinal patterns after using Kolmogorov-Smirnov tests, followed by Bonferroni corrections of *p*-values. Variation explained by the model was determined using the *model.avg* function of the R package *MuMIn* v.1.43.17 [50]. To visualise the richness along the elevational gradient, we used the R package *ggplot2* v.3.3.5 [51].

#### Elevational species turnover.

The variation in species composition along the elevational gradient (β-diversity) was evaluated using Baselga’s incidence-based dissimilarity measures for multiple sites [52,53]. Multiple site beta diversity provides information on the spatial heterogeneity of community assemblages [53] and can be partitioned into turnover and nestedness components. We then evaluated elevational β-diversity across altitudinal bands for all MOTUs’ data using the Sørensen index. Total dissimilarity, turnover and, nestedness were computed through pairwise comparisons between all nine elevations and plotted with heat maps to represent β-diversity between elevations. This procedure was repeated for two data subsets: insects identified at species level and insects identified at genus level. Spearman’s rank correlations were used to test the resemblance of total dissimilarity, turnover and nestedness between species and genus results [25]. The β-diversity calculations were performed using the *betapart* package v.1.5.4 [53]. All analyses were conducted in the R statistical software v.4.3.2 [41]. The complete dataset and R script are available as supporting information (S1 Files & S2).

## Results

### Insect diversity and community composition

**Fig 1:**
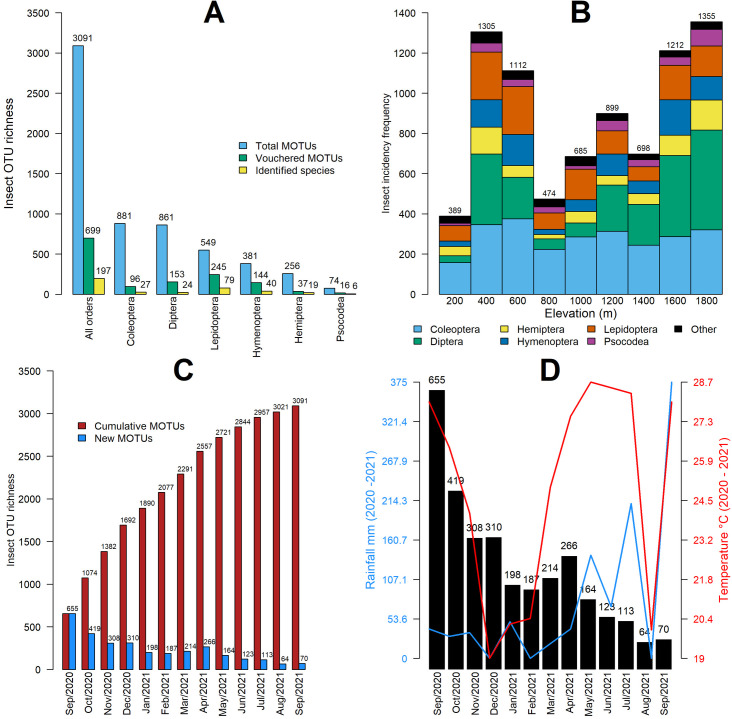
A) Insect richness (total number of MOTUs), backed by vouchers and identified species sampled at El Cielo study site, showing the overall quantity and distribution among the six most common orders. B) MOTU distribution of the six most common insect orders sampled at the nine altitudinal bands of El Cielo study site. The remaining 10 insect orders are clustered in the “Other” category. Numbers on top of each bar represent the overall insect incidence frequency in that altitudinal band. C) Cumulative and new MOTUs found in the 13 sampling months. Numbers on top of each bar represent the overall insect species richness in that month. By the fourth month (December 2020) over 50% of the total MOTUs had been sampled (1692 out of 3091). D) Insect species richness (number of MOTUs) found in the 13 sampling months overlaid with a climograph. Black bars stand for insect richness on each month, on top of each bar its numeric richness value is displayed. The red line represents the temperature (°C) across the 13 sampling months (notice the abrupt rise in the summer of 2021, matching the decrease in insect richness), while the blue line stands for the rainfall precipitation (mm) across the 13 sampling months.

A total of 3091 MOTUs were identified from the 117 samples collected along the El Cielo elevational gradient. On average, each trap caught 560 ± 199 SD MOTUs, with 2.63 ± 3.16 specimens per MOTU. Rarefaction/extrapolation curves showed similar trends of inventory completeness (S2 Fig), with a sample coverage of 80%. Of the identified MOTUs, 699 (22.6%) were matched to voucher specimens, 485 (15.7%) were identified to genus level, and 197 (6.4%) were identified to species level (Fig 1A). The six most common orders were Coleoptera (881 MOTUs), Diptera (861), Lepidoptera (549), Hymenoptera (381), Hemiptera (256), and Psocodea (74), while the remaining ten orders (Archaeognatha, Blattodea, Dermaptera, Mantodea, Mecoptera, Phasmatodea, Psocoptera, Strepsiptera) only accounted for 89 MOTUs (Fig 1B).

The total insect MOTU density per trap per monthly sample (n = 117) averaged 70 ± 39 MOTUs (mean ± standard deviation). By the fourth month of sampling, over 50% of the cumulative total number of MOTUs had been sampled (1692 out of 3091; Fig 1C). Of the total MOTUs, 51% (1580 out of 3091) were singletons, occurring only in one elevation or sampling month (S3 Fig). Among the ten most common MOTUs across the altitudinal gradient, only two had BOLD and NCBI matches for Mexico (S1 Table), and only three taxa throughout the sampling period had sequences previously reported for Mexico (S2 Table).

### Environmental factors shaping insect assemblages

The best-fit GLM for insect incidence frequency, after model simplification, included two environmental variables and one interaction (Table 1; model McFadden pseudo-*R*^*2 *^= 0.351). Annual Mean Temperature explained nearly a third of all insect incidence frequency variation across El Cielo, with decreased insect incidence as temperature increased. Meanwhile, tree richness explained just 2% of insect variation: insect incidence frequency decreased with increasing tree richness. Tree richness and the interaction between both environmental predictors explained less than 5% of insects’ diversity patterns variation.

**Table 1:** Effects predicted by the simplest generalized linear model of El Cielo insect incidence frequency given its environmental factors.

Source	Slope	Standard Error	McFadden pseudo-*R*^*2*^	*p*
Tree richness	−0.856	0.162	0.021	<0.0001
Annual Mean Temperature (BIO1)	−0.51	0.075	0.309	<0.0001
Tree richness × BIO1	−0.04	0.008	0.022	<0.0001

Multivariate abundance analysis showed that 52% of the variation in co-occurrence patterns among insect communities across the elevational gradient of El Cielo can be explained by the contemporary temperature and rainfall (2020–2021) during the 13-months of sampling (Fig 1D). In contrast, only 21% of the variation can be explained by historical temperatures and precipitation (1991–2020) (S3 Table). Insect incidence frequency matched the seasonal changes in temperature and rainfall (S4 Fig). Coleoptera increased with rising temperatures but decreased at the beginning of the rainy season in August – October [27]. In contrast, Diptera and Lepidoptera exhibited the opposite trend, showing a decline during the highest temperatures and slightly increasing with the onset of rains.

### Elevational diversity patterns of insect orders

**Fig 2:**
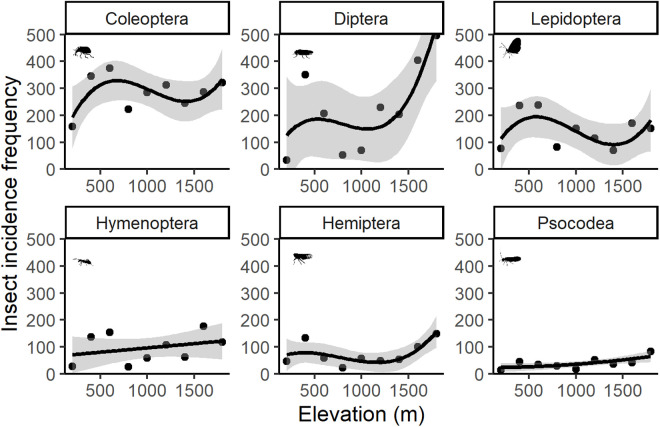
Altitudinal diversity patterns of the six most common insect orders sampled at El Cielo study site. Each point represents a Malaise trap (n = 9). Solid black lines stand for the best-fitted model prediction. Grey shading represents 95% confidence intervals.

The best-fitted model that explained the relationship between MOTU richness and elevation varied across the six orders (Fig 2), ranging from a linear increase with elevation for Hymenoptera to an exponential increase for Psocodea and humped-shaped elevation patterns for Coleoptera, Diptera, Hemiptera, and Lepidoptera (Table 2).

**Table 2:** Model outputs for regression models of insect order richness along the altitudinal gradient of El Cielo.

Insect order	Model	Altitudinal richness pattern	Lowest AICc of compared models	ΔAICc of the chosen model	Model residuals’ *p*-Shapiro	ωAIC (model weight)	Orders with different patterns (K-S test *p* < 0.01)
Coleoptera	Cubic	Humped-shaped	134.73	0	0.5355	1	Hymenoptera, Hemiptera, Psocodea
Diptera	Cubic	Humped-shaped	373.08	0	0.4578	1	Psocodea
Lepidoptera	Cubic	Humped-shaped	158.34	0	0.6235	1	Psocodea
Hemiptera	Cubic	Humped-shaped	109.68	0	0.681	0.58	Coleoptera, Psocodea
Hymenoptera	Linear	Linear increase	169.98	0	0.3485	0.51	Coleoptera, Psocodea
Psocodea	Exponential	Exponential increase	61.30	0	0.591	0.36	Coleoptera, Diptera, Lepidoptera, Hemiptera, Hymenoptera

### Elevational species turnover

**Fig 3:**
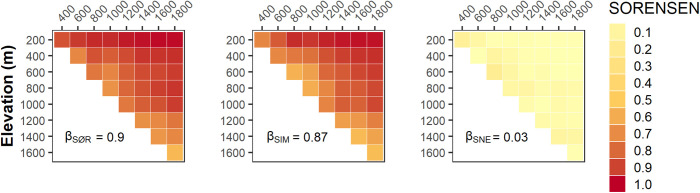
Heat maps displaying species β-diversity (n = 3091 MOTUs), measured with Sørensen index, along El Cielo gradient between elevational levels. Total β-diversity (left, β_SØR_) has been partitioned into turnover (middle, β_SIM_) and nestedness (right, β_SNE_). β-diversity (0 → 1) near 1 means insect assemblages have different MOTUs between the compared elevations, while 0 means having the same genera. Turnover (0 → 1) close to 1 means the dissimilarity is driven only by the replacement of MOTUs along the elevational gradient (i.e., without changes in richness). Conversely, nestedness close to 1 means the dissimilarity is driven only by differences in richness (poorest communities hosting a subset of MOTU present in the richest one).

Insect MOTU dissimilarity was high along the El Cielo elevational gradient (mean β_SØR_ = 0.9). Turnover contributed much more than nestedness (β_SIM_ = 0.87 > β_SNE_ = 0.03; Fig 3), indicating that hardly any insect MOTUs were shared between the top and the base of the mountain. Even when β-diversity was partitioned by insect order, turnover had a greater impact on insect community composition than nestedness (S5 Fig). For insects identified at the species-level (n = 197) and genus-level (n = 485), the patterns remained the same: high dissimilarity with turnover contributing more than nestedness for species (β_SØR_ = 0.9, β_SIM_ = 0.87, β_SNE_ = 0.03) and genera (β_SØR_ = 0.87, β_SIM_ = 0.82, β_SNE_ = 0.05). Spearman’s rank correlations revealed differences between the pairwise of species-level (n = 197) and genus-level (n = 485) community composition, indicating that there was no 1:1 correlation between species-level and genus-level values for dissimilarity (ρ = 0.18, *p* = 0.299), turnover (ρ = 0.21, *p* = 0.31) and nestedness (ρ = 0.36, *p* = 0.032).

## Discussion

Integrating novel methodologies, such as metabarcoding, into long-term studies can provide extensive and valuable ecological insights into insect communities [54]. To the best of our knowledge, this study represents the most comprehensive assessment of elevational fluctuations with insect metabarcoding-derived data within the Mexican Transition Zone, evaluating the seasonal and spatial influence of environmental factors on the altitudinal patterns of a diverse array of insect orders. As part of our assessment, we hypothesized that insect diversity would be explained either by the mid-domain effect or by environmental predictors such as temperature and rainfall, follow hump-shaped patterns, and be driven by both species turnover (replacement) and nestedness (species gains and losses). Insect species richness variance in our study was best explained by temperature and vegetation composition, four out of the six major insect orders followed hump-shaped diversity patterns, and turnover explained 29 times more insect community dissimilarity than nestedness.

### Insect diversity and community composition

Our 80% sample coverage of Malaise-caught insects along the altitudinal gradient aligns with usual levels reported in previous metabarcoding studies of tropical insects [55,56]. Out of the 3091 insect MOTUs encountered along El Cielo elevational gradient, 699 (22.6%) were matched with the species level DNA vouchers, however only 197 (6.37%) were completely identified to species level. This ratio underscores the lack of comprehensive resources and DNA voucher material for adequately identifying arthropods in the region. As the number of insect species is rapidly declining [57] the risk of species disappearing before they have been formally described [58] is a pressing concern. Although many species in our study may have already been described, they still lack accessible genetic information to be detected in DNA-based inventories. Greater efforts for developing and expanding the barcoding databases linked to museum-quality vouchers are needed in the region, especially for understudied and hyperdiverse insect taxa.

If a species was caught at every elevation (9 altitudinal bands) in every sampling event (13 months) then it would have 117 records, yet no insect came close to this number: the range was between 1–37 records per species. The only four species that had above 30 records were an unknown hemipteran (n = 37), a bristletail from the family Machilidae (n = 36), a fly from the genus *Pseudolycoriella* (n = 31) and a booklice from the family Ptiloneuridae (n = 31). All four had their incidence frequency peak in the oak-pine forest at 1200–1600 m of elevation. Yet the overall incidence frequency per order peaked at lower elevations of ~ 500 m, corresponding to the tropical forest. The peaks in species richness around 500 m for orders such as Diptera and Lepidoptera are consistent with the results of previous studies [4,6], which report an increase in incidence frequency and species richness along elevational gradients up to 500 m, after which both begin to decline.

### Environmental factors shaping insect assemblages

Temperature is a well-known environmental predictor of mountain biodiversity [11,12], especially for small-bodied animals like insects that are highly susceptible to temperature changes [15,59–61]. In this study, temperature (taken as Annual Mean Temperature) explained the highest variation in insect incidence frequency (Table 1), underscoring its importance in driving species richness and abundance across the elevations. Elevation, often considered a proxy for temperature, was excluded from the analysis due to its strong covariance with temperature.

Current temperature and rainfall seasonality fluctuations accounted for more than half of the total variation in insect incidence frequency observed during the 13-month sampling period. In contrast, only 21% of the variation was explained by historical temperature and rainfall averages over the past 30 years (S3 Table). This suggests that recent climate fluctuations may significantly impact insect incidence frequencies more than long-term historical trends. Since the mid-20^th^ century, temperatures in mountainous regions have been rising faster than the Northen Hemisphere average [62], with more pronounced warming trends than the global average [63]. Unusually high temperatures and low precipitation rates have also become more frequent [64]. For example, China’s Mt. Gongga has experienced more frequent climate anomalies after the year 2000 than in previous decades [64]. These changes in abiotic conditions have driven many montane insect species to shift their ranges upwards, with Lepidopterans [65] and Hymenopterans [66] being among the most affected. In El Cielo, a notable temperature and rainfall fluctuation occurred near the end of the sampling period, coinciding with a decline in the number of new insect MOTUs captured in the Malaise traps (Fig 1D). Thus, the recent climatic changes in our study area likely explain the observed fluctuations in insect community incidence frequency across the altitudinal gradient, highlighting the significant effect of contemporary climate seasonality.

Vegetation composition, measured here as tree species richness, acted as a biotic filter for herbivore assemblages like Lepidopteran larvae [67] and phytophagous beetles [24,28], explaining 2.1% of the variation in insect incidence frequency (Table 1). The interaction between tree species richness and temperature accounted for an additional 2.2% variation. Although these effects are smaller compared to temperature alone, their ecological significance should not be dismissed, as small differences can accumulate over many generations, leading to substantial long-term effects [68]. Current tree species diversity in El Cielo may be acting as a biotic filter, since the last developmental stage of the biotic assembly in the Mexican Transition Zone during the Pliocene – Pleistocene [26]. Further research should evaluate the contributions of biotic filtering and niche differentiation in El Cielo insect community structure.

### Elevational diversity patterns of insect orders

As hypothesized, species richness exhibited a humped-shaped pattern for all insect orders except Hymenoptera and Psocodea, which showed a linear increase and an exponential increase, respectively. Previous studies have reported unimodal richness peaks for insects [4,6,14]. The mid-domain effect explains that humped-shaped richness patterns arise from the random overlap of species distributions across mountains [11,21]. In our study, Coleoptera, Diptera, Hemiptera and Lepidoptera richness peaked at around 500 m, near the ~ 800 m boundary between tropical and cloud forests [28]. The overlap between the upper limits of lowland habitats and the lower limits of highland habitats may explain the mid-altitude richness peaks along the elevational gradient [69].

Insects exhibit diverse lifestyles that enable them to thrive in a wide variety of environments [70]. In our sampling we predominantly caught flying insects and soil-dwelling insects. Life history may explain the elevational pattern observed at the study area: we recoded higher insect incidence frequencies in altitudes with lower or moderate tree richness (10–12 tree species per plot), which quickly declined in areas with greater tree species richness. In forests with few tree species, insect communities often exhibit higher rates of herbivory, particularly when most species have specialized diets (i.e. oligophagous herbivory: insects that are host-specific to trees within a single taxonomic family or genus [71]). Additionally, the herbivorous larval diets of flying insects, such as Lepidoptera, tend to be more specialized in tropical forests when compared to temperate forests [72,73]. Therefore, in our tropical elevational gradient less diverse forests might experience higher rates of herbivory by flying insects (Lepidoptera), which in turn result from a higher insect incidence frequency recorded at these altitudes. Nevertheless, further research is needed to better understand the influence of tree species richness, along other biotic and abiotic factors, on insect diversity along this gradient.

Biotic factors, such as biological interactions, and abiotic predictors, like seasonality or extreme weather events, can shape the elevational patterns of insect diversity [74–76]. While some insect groups show consistent declines of diversity with elevation [75], others, like ants, may show different patterns specially if extreme weather conditions like tropical storms, occur [76]. Moreover, these patterns can shift with latitude, with increased number of species and individuals in mid-latitudes potentially driven by shorter growing seasons [74]. It is important to note though, that all biological explanation, will be biased to the sampling technique used. Here, we captured mobile understory insects across the elevation gradient. Other techniques will represent groups with different life histories – such as canopy, soil-dwelling or flightless insects – and may yield contrasting diversity patterns.

### Elevational species turnover

Insects in the El Cielo elevational gradient are stratified by altitude, as shown by the high species turnover contribution to the overall dissimilarity of the community composition. High elevational turnover can be expected in some tropical insects as demonstrated by studies of individual taxa such as ants in Central America and Papua New Guinea [14,15], beetles in Mexico [17,23] and moths in Costa Rica and Australia [4,6]. Up to 30% of the insect community turnover across the mountain was explained by temperature: a higher insect incidence frequency was found at higher temperatures at the base and in the centre of the elevational gradient than at the lower temperatures at the summit. It is known that temperature change strongly correlates with altitude, and it is a fundamental abiotic driver of community diversity and composition [10,11]. As temperature is intrinsically linked to elevation itself [6,10], altitude may be shaping insect biodiversity composition across the elevational gradient.

Turnover was highly significant across El Cielo at both species and genus levels. This indicates that not only insect species, but also insect genera community composition differs between the base and the summit of the mountain. Similar patterns across different taxonomic scales have been reported for other hyperdiverse invertebrate taxa, such as molluscs [25]. However, genus level heterogeneity does not mirror species-level heterogeneity, meaning that turnover patterns at these two taxonomic levels differ across the insect communities of El Cielo elevational gradient. In other invertebrate groups species-level heterogeneity is reflected at higher taxonomic levels (e.g. genus and family levels [25]). However, in such cases, the geographical scale of the studies was considerably larger than our study site (e.g. previous research analyzed data from four different oceans [25]).

When considering a single mountain, such as El Cielo, high β-diversity may arise from variation among species within a few genera. While some genera may be present across multiple elevations, their constituent species could be unique to specific altitudes, leading to greater dissimilarity throughout the mountain. This pattern is exemplified by the ant genus *Camponotus* (with four MOTUs identified to species level) which was recorded between 200–1600 m: one species was restricted to 200 m, another to 600 m, a third to 1600 m, and the fourth occurred between 600–1400 m. A similar trend was observed for the phorid fly *Megaselia*, found between 400–1600 m, with one of its three identified species exclusively recorded at 1400 m. Also, the ant genus *Pheidole* was recorded between 200–1000 m, with two out of its three identified species restricted to 200 m and 600 m, respectively.

Finally, in previous studies [25] samples sizes compared across different taxonomic levels differed by no more than 50%. For instance, prior works [25] compared 163 mollusc species with 75 mollusc families. In contrast, our genus-level dataset (n = 485) was almost 2.5 times larger than our species-level dataset (n = 197). The presence of speciose genera, differences in geographic scales, and the disparity in sample sizes between genera and species could all be contributing to the observed heterogeneity along El Cielo elevational gradient. Therefore, further research is needed to fully elucidate this intriguing pattern.

## Conclusions

Our study is the first to provide a comprehensive assessment of insect diversity along an altitudinal gradient in the Mexican Transition Zone using metabarcoding. This significant gap in taxonomic knowledge for the study area highlights the urgent need for comprehensive reference materials, and voucher specimens, to enable accurate arthropod identification in the region. Humped-shaped diversity patterns reflected the different insect lifestyles. Contemporary climate changes drove species distribution and turnover across altitudes. Differences at both species and genus levels highlighted the complexity of tropical mountain ecosystems. By combining long-term sampling with metabarcoding, we gain valuable insights into the year-round dynamics of insect activity, revealing temporal fluctuations and interactions within these communities. Furthermore, this research serves as a foundation for future long-term monitoring efforts, contributing to the development of more robust frameworks for ecological assessment and biodiversity conservation.

## Supporting information

Fig S1 Map of Malaise traps spaced by approximately 200 m a.s.l. along the El Cielo elevational gradient, Tamaulipas, México. Elevation is shown as a grayscale gradient. (TIF)

Fig S2 (A) Accumulation curves of insects MOTUs for each of the locations (nine elevation sites separated by by 200 m a.s.l., Curves were generated using incidence frequency data. Points represent total observed insect order richness values, while solid lines are interpolation (before the points) and extrapolation (after the points) curves with 95% confidence intervals (shaded areas). (B) Coverage-based rarefaction curves of insect orders with 95% confidence intervals (shaded areas) for nine altitudinal bands in El Cielo gradient. (C) Sample-completeness rarefaction curves with respect to sample size (number of sampled months) for nine altitudinal bands in El Cielo gradient.
(TIF)

Fig S3 (A) The proportion of occupied altitudinal bands (n = 9) by all insect species (MOTUs = 3091) across El Cielo elevational gradient and (B) frequency of occurrence of all insect MOTUsin the whole sampling period (n = 13 months). For details of the most abundant insects across the elevational gradient and the sampling period see S1 and S2 Tables.
(TIF)

Fig S4 Seasonality fluctuation patterns of the six most abundant insect orders sampled at El Cielo study site. Each point represents the insect abundance collected in all the Malaise traps per month (n = 13). Solidh black lines stand for the best-fitted model prediction. Grey shading represents 95% confidence intervals. Rainy season usually occurs from August – October (González-Medrano, 2005).
(TIF)

Fig S5 Altitudinal β-diversity patterns of the six most abundant insect orders sampled at El Cielo study site. Each point represents the mean β-diversity value of the pairwise comparisons among all the Malaise traps per altitudinal band (n = 9). Solid black lines stand for the best-fitted model prediction. Grey shading represents 95% confidence intervals. For display purposes, the y-axis scales for the total dissimilarity and turnover measurements were set to values ranging from 0.4–1, while nestedness was set to 0–0.15.
(TIF)

Table S1 Information about the top ten insect species (MOTUs) with the highest proportion of occupied altitudinal bands. The first two species occupied 8/9 altitudinal bands, while the other eight occupied 7/9 altitudinal bands. The information provided corresponds to the best-matched BOLD sequence for each insect species.
(TIF)

Table S2 Information about the top ten insect species (MOTUs) with the highest frequency of occurrence across the entire sampling period. The first four species appeared in all of the sampling months (13), while the following six appeared in 12/13 months. The information provided corresponds to the best-matched BOLD sequence for each insect species.
(TIF)

Table S3 Monthly mean temperature and rainfall: historical (1990–2020) and contemporary (2021) records. (TIF)

File S1 Databases. (XLSX)

File S2 R code. (TXT)
