# Supplementary figures and images for: Unravelling high insect diversity and community turnover along a tropical-temperate elevation gradient: A metabarcoding approach
Source: PLoS One. 2025 Jul 17;20(7):e0327884. doi: 10.1371/journal.pone.0327884 (PMC12270123; doi:10.1371/journal.pone.0327884)

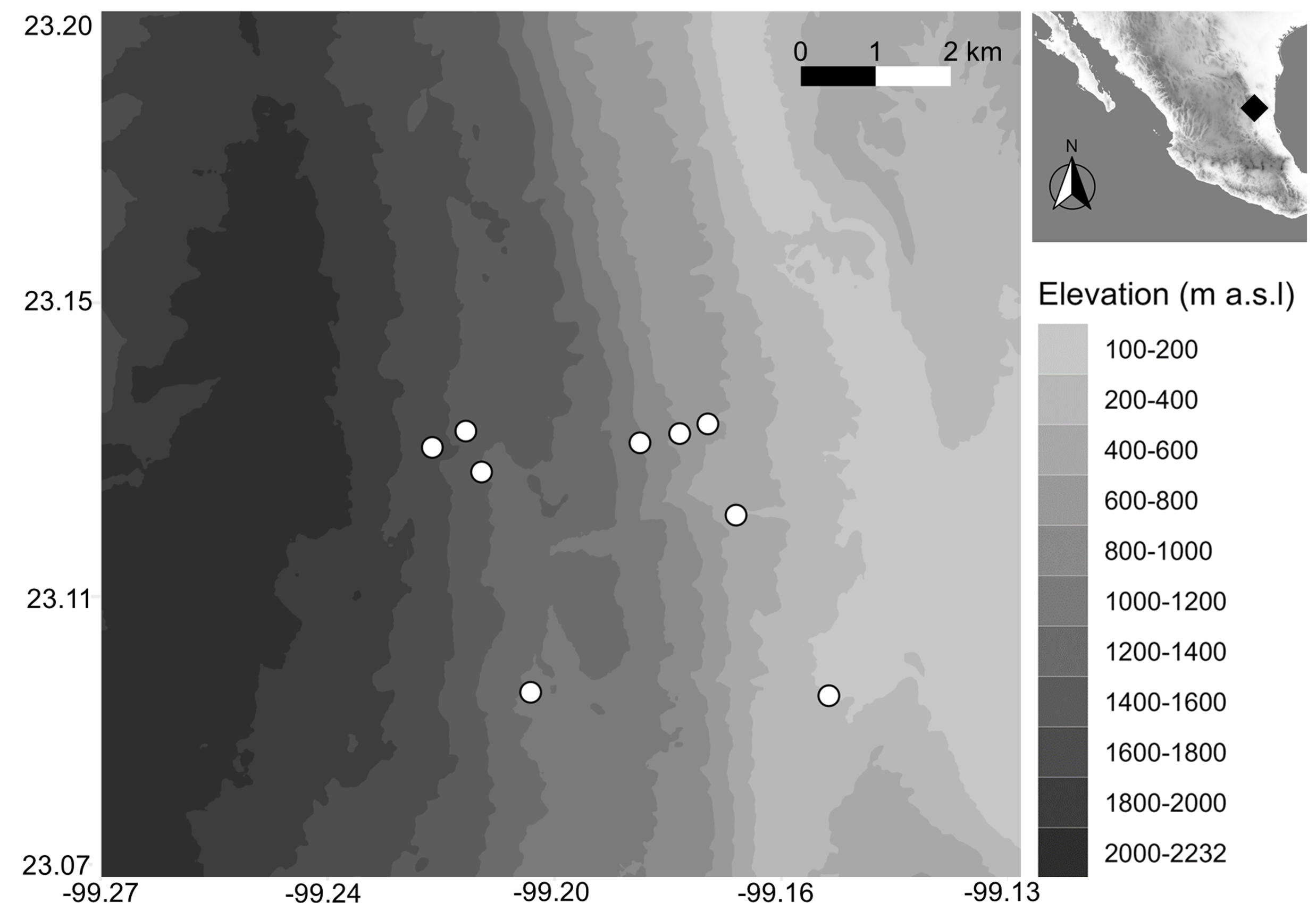

Supplement: Fig S1 — (TIF) [file pone.0327884.s001.tif]

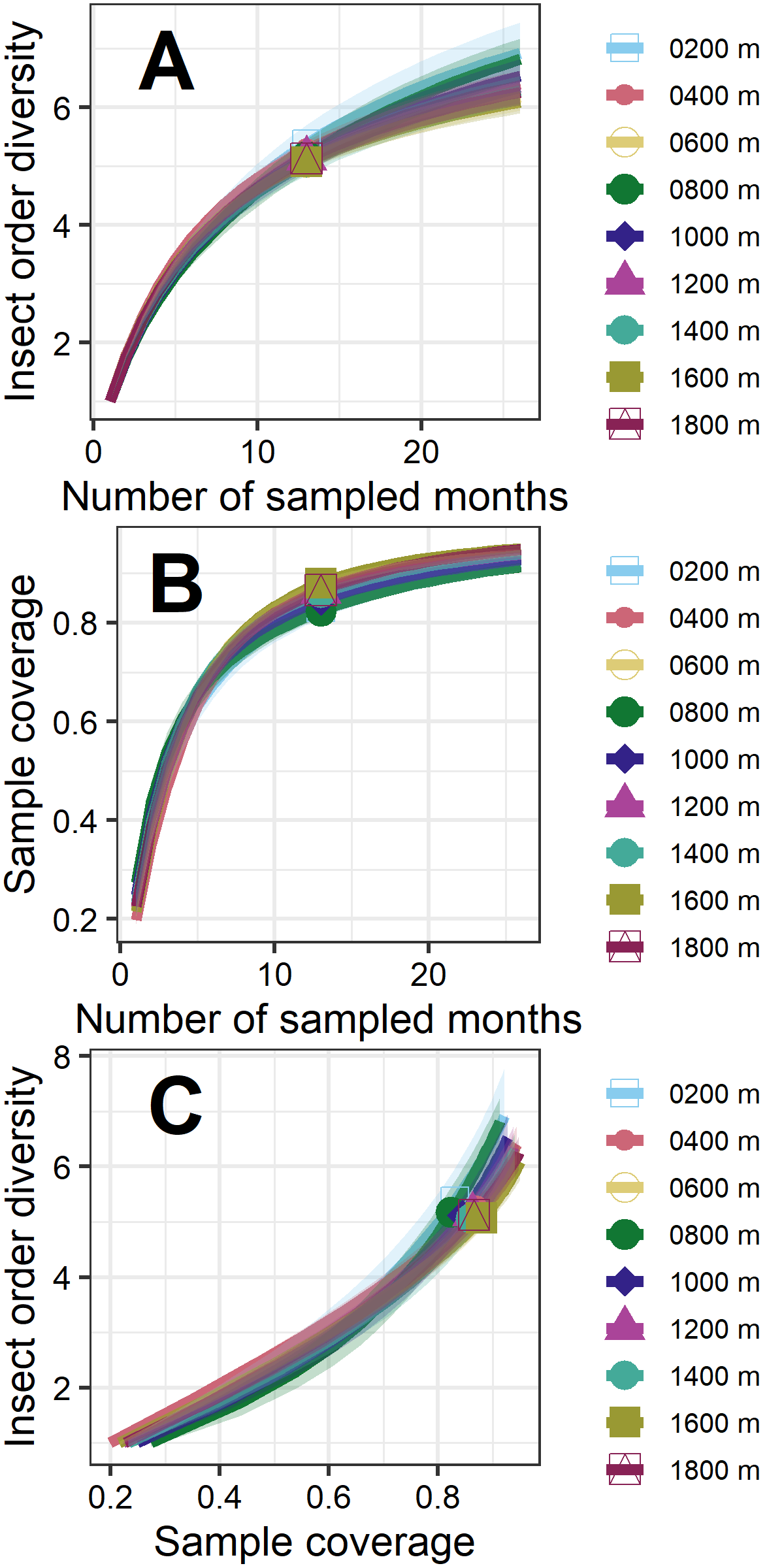

Supplement: Fig S2 — Points represent total observed insect order richness values, while solid lines are interpolation (before the points) and extrapolation (after the points) curves with 95% confidence intervals (shaded areas). (B) Coverage-based rarefaction curves of insect orders with 95% confidence intervals (shaded areas) for nine altitudinal bands in El Cielo gradient. (C) Sample-completeness rarefaction curves with respect to sample size (number of sampled months) for nine altitudinal bands in El Cielo gradient. (TIF) [file pone.0327884.s002.tif]

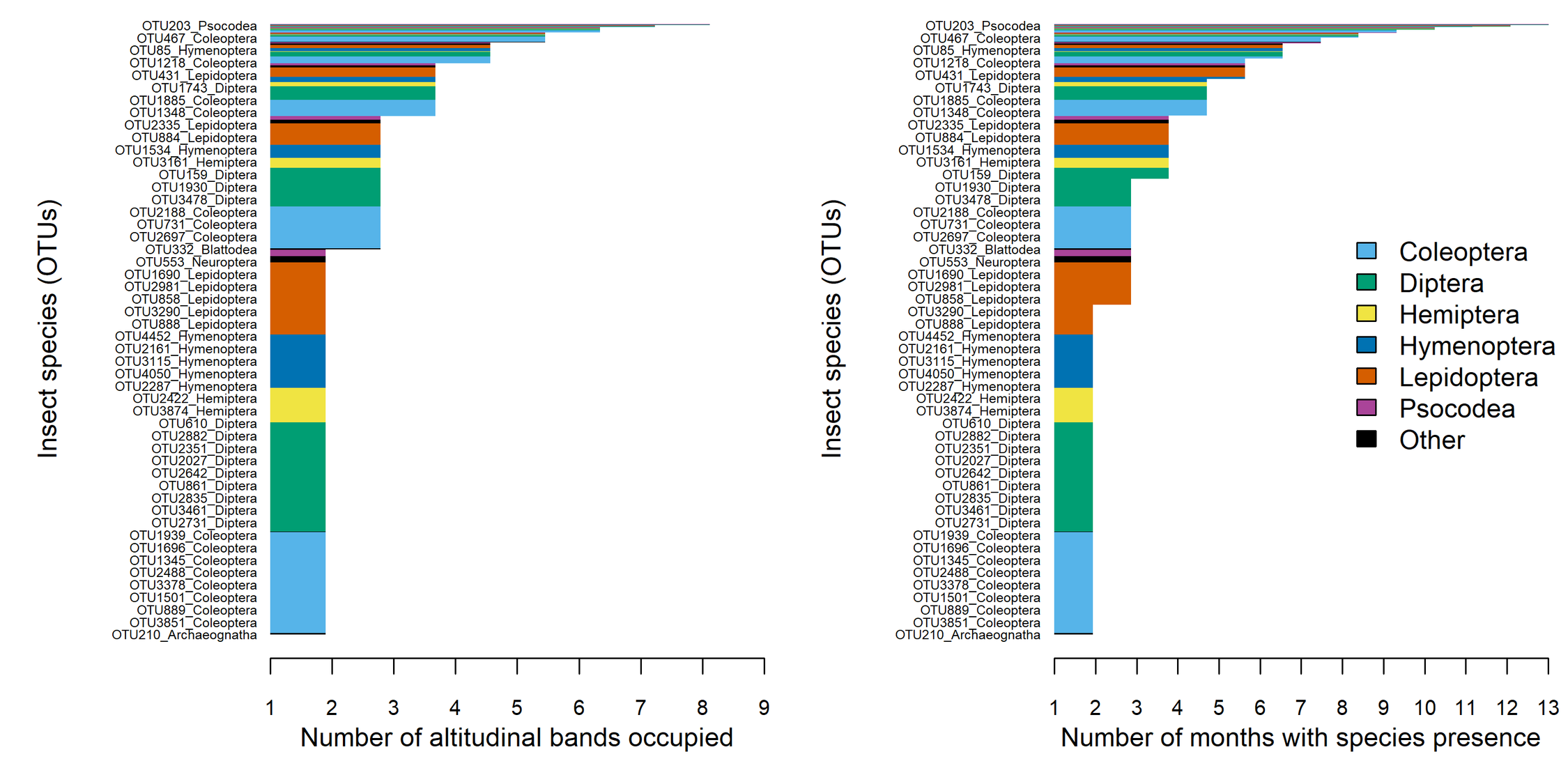

Supplement: Fig S3 — For details of the most abundant insects across the elevational gradient and the sampling period see S1 and S2 Tables. (TIF) [file pone.0327884.s003.tif]

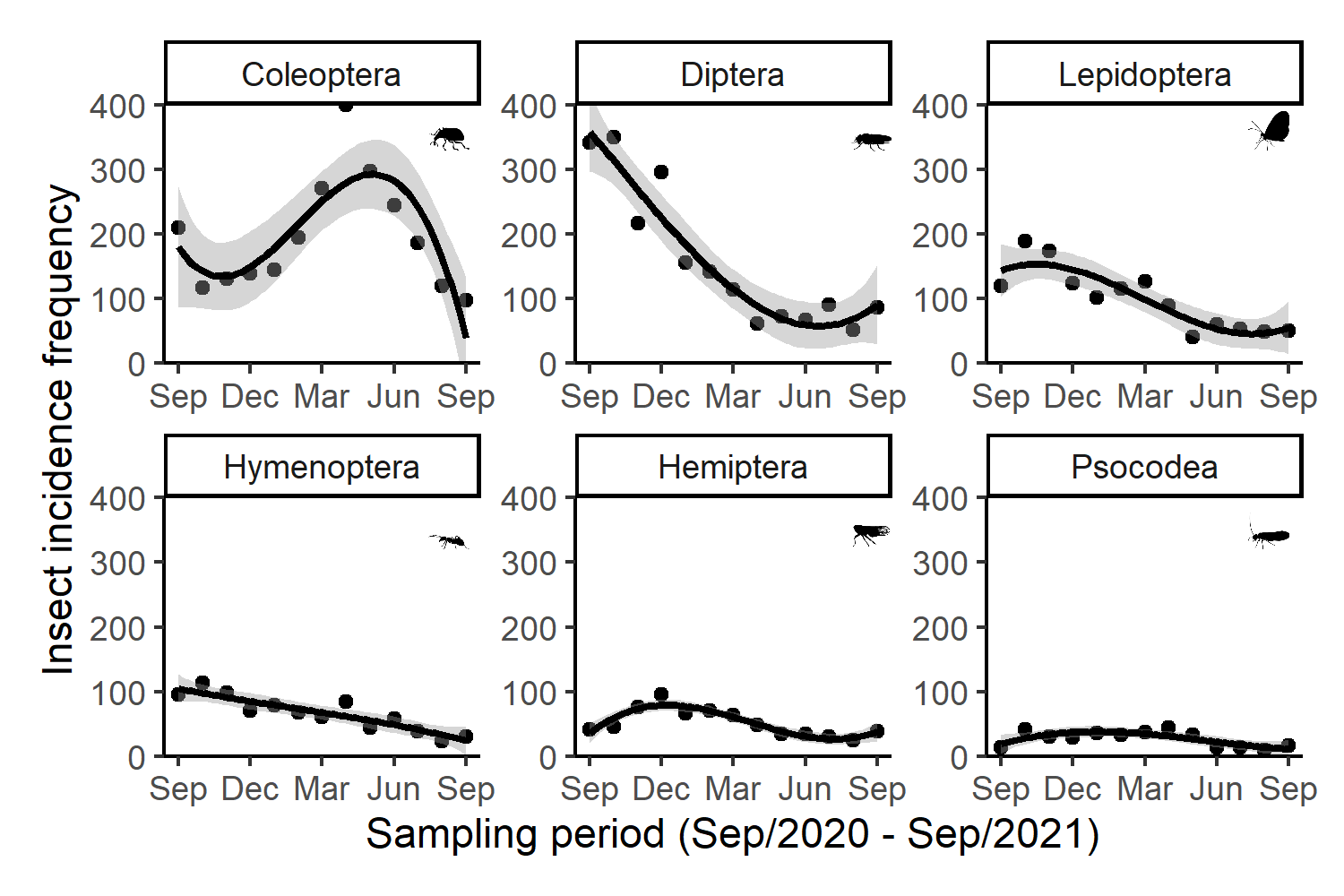

Supplement: Fig S4 — Each point represents the insect abundance collected in all the Malaise traps per month (n = 13). Solidh black lines stand for the best-fitted model prediction. Grey shading represents 95% confidence intervals. Rainy season usually occurs from August – October (González-Medrano, 2005). (TIF) [file pone.0327884.s004.tif]

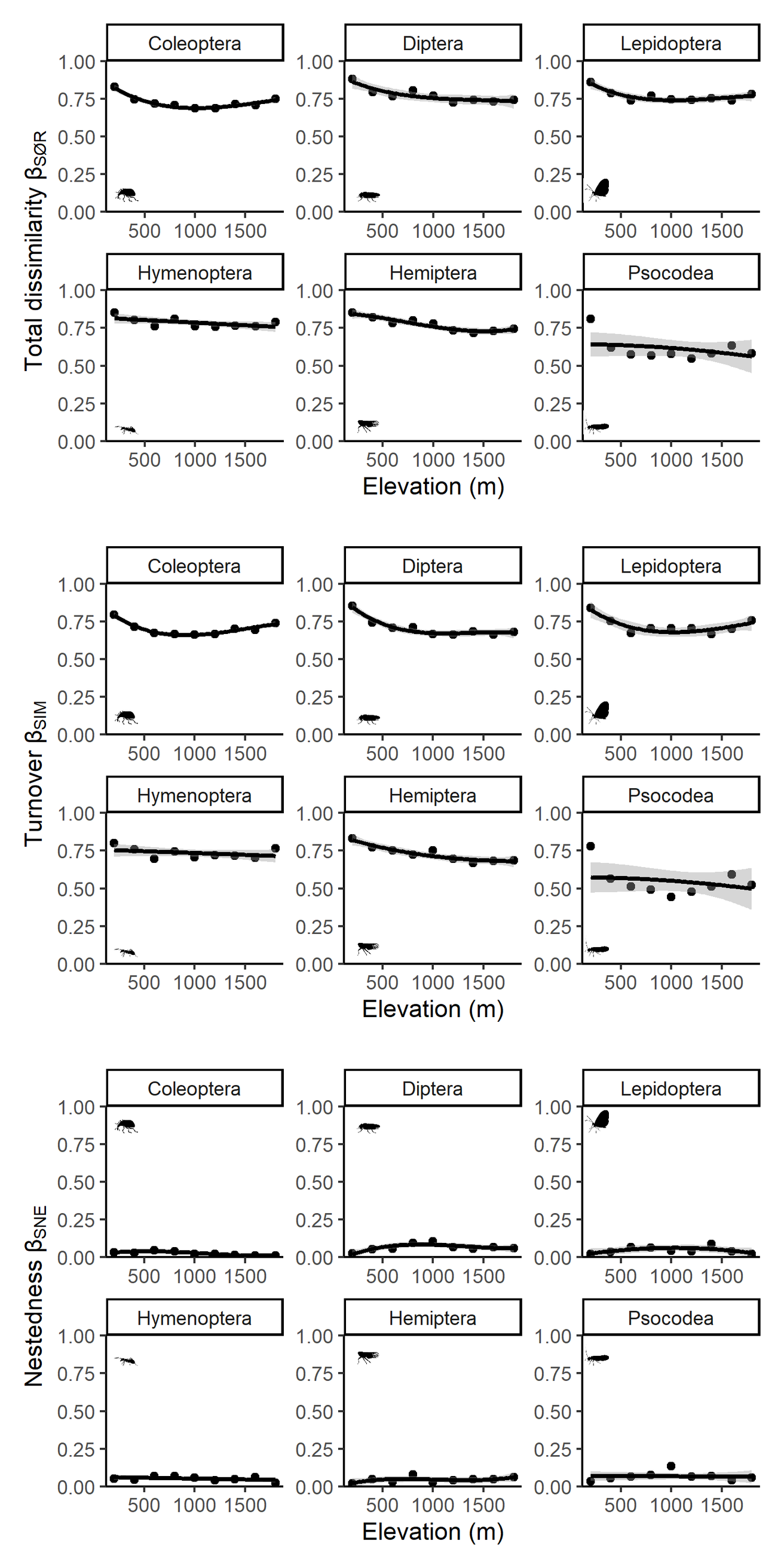

Supplement: Fig S5 — Each point represents the mean β-diversity value of the pairwise comparisons among all the Malaise traps per altitudinal band (n = 9). Solid black lines stand for the best-fitted model prediction. Grey shading represents 95% confidence intervals. For display purposes, the y-axis scales for the total dissimilarity and turnover measurements were set to values ranging from 0.4–1, while nestedness was set to 0–0.15. (TIF) [file pone.0327884.s005.tif]

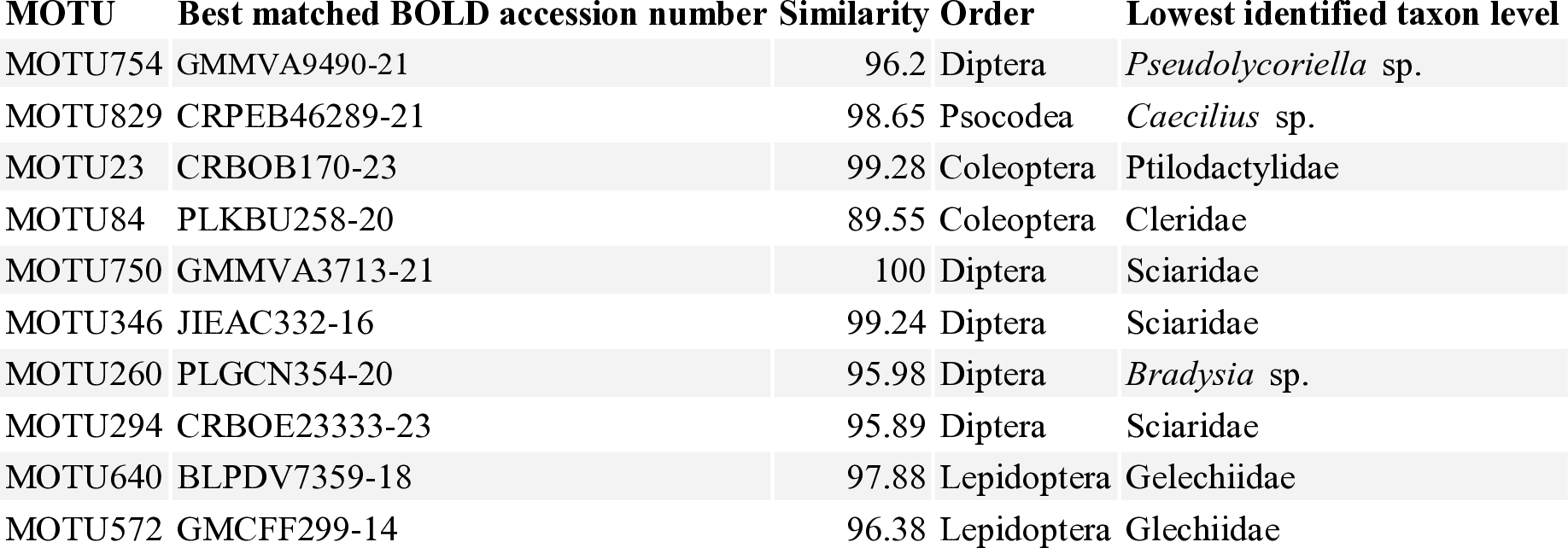

Supplement: Table S1 — The first two species occupied 8/9 altitudinal bands, while the other eight occupied 7/9 altitudinal bands. The information provided corresponds to the best-matched BOLD sequence for each insect species. (TIF) [file pone.0327884.s006.tif]

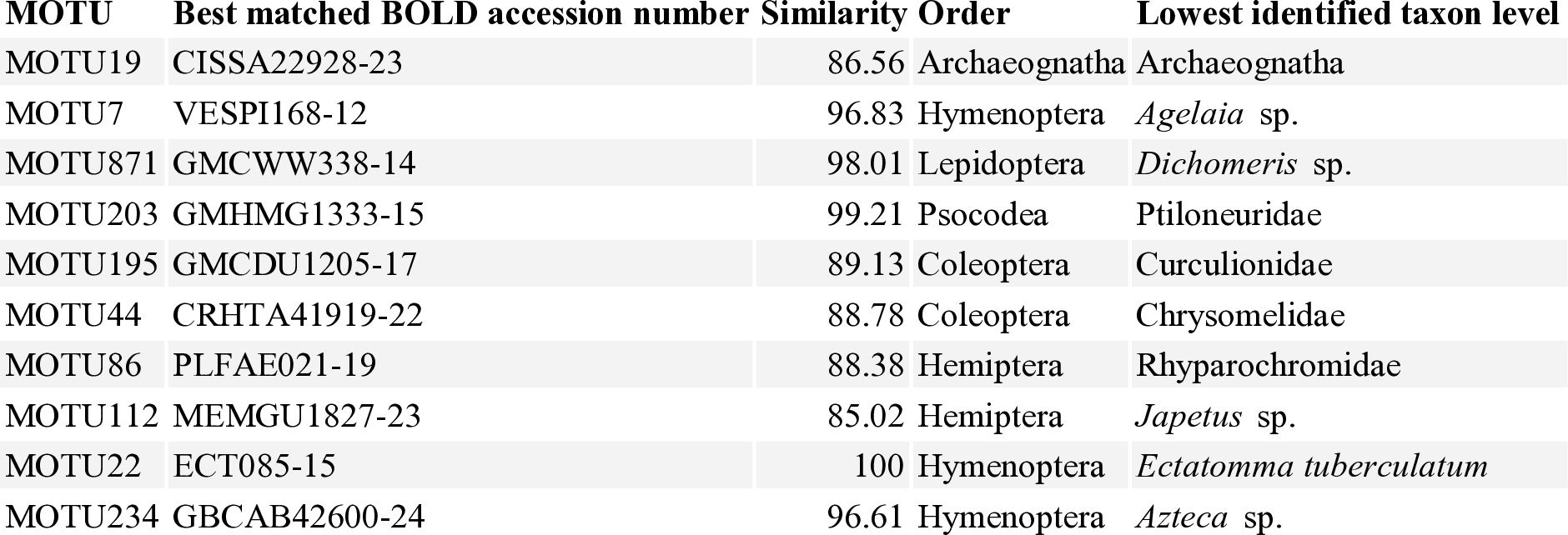

Supplement: Table S2 — The first four species appeared in all of the sampling months (13), while the following six appeared in 12/13 months. The information provided corresponds to the best-matched BOLD sequence for each insect species. (TIF) [file pone.0327884.s007.tif]

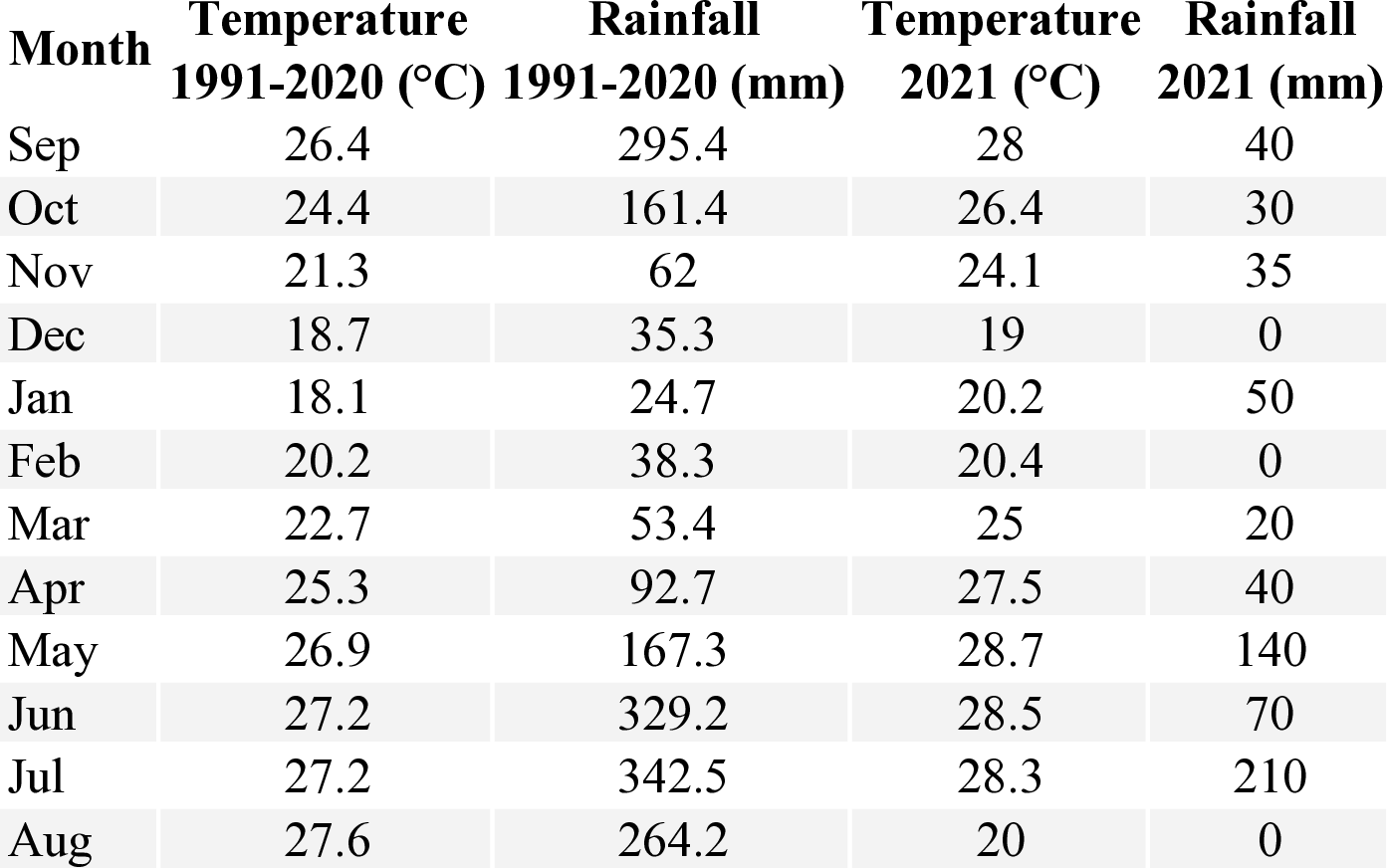

Supplement: Table S3 — (TIF) [file pone.0327884.s008.tif]
